# P-233. Detectable coronary artery calcium score among people living with HIV and regional variations: a systematic review and meta-analysis

**DOI:** 10.1093/ofid/ofaf695.455

**Published:** 2026-01-11

**Authors:** Jose Luis Paredes, Danai Bemplidaki, Alvaro Matos Arana, Aniruddha Hazra

**Affiliations:** Advocate Illinois Masonic Medical Center, chicago, IL; The Health Research Unit Zimbabwe, Biomedical Research and Training Institute, Harare, Zimbabwe, chicago, Illinois; Ascension St Joseph, Chicago, Illinois; University of Chicago, Chicago, IL

## Abstract

**Background:**

People living with HIV (PLWH) experience up to twofold increased risk of cardiovascular disease compared to the general population. The coronary artery calcium (CAC) score offers a non-invasive and quantifiable assessment of subclinical coronary artery disease (CAD), making it a valuable tool for early detection and risk stratification in this high-risk population.

**Methods:**

A systematic review and meta-analysis were conducted to quantify the percentage of detectable CAC among PLWH. Detectable CAC was defined as a CAC >0 based on the Agatston method. CAC scores were further categorized into mild (1–100), moderate (101–399), and high ( >400). Pooled percentages with corresponding 95% confidence intervals (95%CI) were calculated. Subgroup analyses were performed by continent to assess geographic variations in the prevalence of detectable CAC among PLWH.

**Results:**

A total of 56 studies were included, with a pooled percentage of detectable CAC among PLWH of 45.7% (95%CI 36.5-48.2). The pooled percentage with mild CAC was 27.7% (95%CI 22.7-32.9%, N=17), moderate CAC was 9.8% (95%CI 7.7-12.0, N=16) and high CAC 4.4% (.8-6.3%, N=10). Significant regional variations were found in the percentage of PLWH with detectable CAC (p< 0.01): with the highest percentage reported in North America (49.3%, 43.6-55.1, N=35) followed by Asia (42.3%, 95%CI 37.7-48.2, N=5), Europe (42.3%, 95%CI 36.5-48.2, N=12) and Africa (13.4%, 95%CI 9.6-17.6, N=3). A single study from South America reported a pooled percentage of PLWH with detectable CAC of 67.4% (95%CI 50.9-81.4).

**Conclusion:**

Almost half of PLWH had detectable CAC, indicating a substantial burden of subclinical coronary artery disease in this population. The higher prevalence observed in North America highlights the importance of targeted preventive strategies, including lifestyle interventions and pharmacologic therapies. The scarcity of data from South America, Oceania and Africa highlights a critical need for further research in these underrepresented regions.

**Disclosures:**

Aniruddha Hazra, MD, Gilead Sciences: Advisor/Consultant|Gilead Sciences: Grant/Research Support|ViiV Healthcare: Advisor/Consultant

